# Computed tomography findings in patients with pulmonary tuberculosis and diabetes at an infectious disease hospital in China: a retrospective cross-sectional study

**DOI:** 10.1186/s12879-023-08386-7

**Published:** 2023-06-27

**Authors:** Qianwen Yang, Rongping Zhang, Yan Gao, Chaoxin Zhou, Weifang Kong, Wang Tao, Guojin Zhang, Lan Shang

**Affiliations:** 1 Department of Radiology, Sichuan Provincial People’s Hospital, University of Electronic Science and Technology of China, Chengdu, China; 2 School of Medicine, University of Electronic Science and Technology of China, Chengdu, China; 3 Department of Radiology, The First People’s Hospital of Liangshan Yi Autonomous Prefecture, Xichang, Sichuan China

**Keywords:** Computed tomography, Tuberculosis, Type 2 diabetes mellitus, Infectious disease, China

## Abstract

**Background:**

This study aimed to investigate the relationship between active pulmonary tuberculosis (TB) and type 2 diabetes mellitus (T2DM) by analysing the clinical features and computed tomography (CT) findings of patients with active pulmonary TB and comorbid T2DM (TB-DM) in the LiangShan Yi regions.

**Methods:**

We collected data from 154 hospitalised patients with TB-DM initially confirmed at an infectious disease hospital in the Liangshan Yi Autonomous Prefecture between 1 and 2019, and 31 December 2021. These were matched by sex and age ± 3 years to 145 hospitalised patients with initially confirmed pulmonary TB without comorbid T2DM (TB-NDM) over the same period. The clinical characteristics of the two groups were analysed separately. Three group-blinded radiologists independently analysed the CT findings and classified them into mild-to-moderate and severe groups. Severe chest CT lesion refers to a lesion that is less diffused or moderately dense and either exceeds the total volume of one lung, a high-density fused lesion greater than one-third of the volume of one lung, or a cavitary lesion with a maximum diameter ≥ 4 cm.

**Results:**

No significant differences were observed in the presentation of clinical features. Regarding the severity of chest CT manifestation, patients with TB-DM had significantly more severe TB than those with TB-NDM (89.61% vs. 68.97%, P < 0.0001). Regarding CT findings, patients with TB-DM had higher proportions of consolidation (79.22% vs. 52.41%, P < 0.0001), cavitary lesions (85.06% vs. 59.31%, P < 0.0001), bronchiectasis (71.43% vs. 31.03%, P < 0.0001), exudative lesions (88.96% vs. 68.28%, P < 0.0001), and fibrous lesions (93.51% vs. 68.97%, P < 0.0001) than patients with TB-NDM. In conclusion, patients with TB-DM have more severe pulmonary TB CT findings than those without. There were no significant differences in the distribution of lesions in the lung lobes between TB-DM and TB-NDM patients.

**Conclusions:**

Among patients hospitalised with pulmonary TB, those with T2DM had more severe findings on chest CT than those without T2DM. However, the clinical presentation was not significantly different.

**Supplementary Information:**

The online version contains supplementary material available at 10.1186/s12879-023-08386-7.

## Background

The LiangShan Yi ethnic minority region in western China has been plagued by poverty for decades, with insufficient medical resources and a high incidence of tuberculosis (TB). The incidence of type 2 diabetes mellitus (T2DM) in economically disadvantaged regions has increased in recent years [1]. Moreover, the proportion of those with undiagnosed diabetes is high in China, a phenomenon that is particularly pronounced in low-income areas [2]. Early diagnosis and adequate control of diabetes are more difficult due to inadequate medical infrastructure, hygienic practices, and cultural education, which in turn lead to an increased risk of TB complications in patients with T2DM. Moreover, early diagnosis and treatment of TB are limited by this lack of resources. Therefore, patients with both TB and diabetes (TB-DM) are at a greater risk for complications of both diseases. The number of TB patients in Liangshan Prefecture is increasing; the reported incidence rate, which was above 100/100,000 for the period 2017–2021, increased by 6.62% from 2017 to 2021 [3].

Chest computed tomography (CT) is an important diagnostic tool for TB. [4–7] The primary signs on CT are local patchy opacities, consolidation, proliferative lesions, tuberculous spheres, tuberculomas, disseminated endobronchial lesions, sclerotic calcifications, and linear opacities. In addition, TB can be classified as mild, moderate, or severe based on chest CT imaging classification criteria [4].

Several studies have investigated the characteristic CT manifestations in patients with TB-DM [8–13]. However, to the best of our knowledge, imaging studies of active pulmonary TB with comorbid T2DM in economically disadvantaged populations have not been reported in the literature.

## Methods

### Aim

This study aimed to investigate the relationship between pulmonary TB and T2DM in a population in the Liangshan Yi Autonomous Prefecture, by analysing the clinical features and CT manifestations of patients with pulmonary TB and comorbid T2DM.

We conducted a retrospective cross-sectional study of adult patients with active pulmonary TB who were hospitalised at the largest infectious disease hospital in Liangshan Yi Autonomous Prefecture, China, between 1 and 2019, and 31 December 2021. The inclusion criteria were as follows: (a) pulmonary TB confirmed by bacteriology (sputum smear or culture) or by polymerase chain reaction of respiratory specimens (sputum, bronchoalveolar lavage, open or percutaneous lung tissue biopsy) and (b) chest CT prior to treatment. The exclusion criteria were as follows: (a) human immunodeficiency virus infection, immunosuppressive therapy, underlying malignancies, or concomitant lung disease (including lung cancer, pneumoconiosis, or other lung infections); (b) CT images of insufficient quality to meet the diagnostic criteria or incomplete data (excessive breathing artefacts, interference from foreign object artefacts, interference from machine artefacts, irregularities in the scope of the scan, etc.); and (c) previous diagnosis or treatment of pulmonary TB.

Patients meeting these criteria were classified into the following two groups:

1. Diabetes mellitus combined with pulmonary TB (TB-DM) group: According to the ‘2020 Chinese Guidelines for the Prevention and Treatment of Type 2 Diabetes Mellitus’, [2] the diagnostic criteria for T2DM were as follows: typical symptoms of diabetes plus random blood glucose level ≥ 11.1 mmol/L, fasting plasma glucose level ≥ 7.0 mmol/L, oral glucose tolerance test 2-h postprandial glucose level ≥ 11.1 mmol/L, or haemoglobin A1c level ≥ 48 mmol/mol.
2. Pulmonary TB without DM (TB-NDM) group: age- (± 3 years) and sex-matched patients with first diagnosed TB admission but no comorbid T2DM.

### Clinical data collection

Clinical information on all patients included sex, age, smoking history, history of long-term heavy drinking, clinical symptoms (cough and sputum, fever, chest pain, weight loss, hemoptysis, headaches, etc.), extrapulmonary TB status, drug-resistant TB status, and specific type of drug resistance were collected from medical records.We used drug susceptibility testing (DST) to detect drug resistance in *Mycobacterium tuberculosis* [14].

### CT scanning and image evaluation

All enrolled patients underwent chest CT as part of screening for pulmonary TB. Three radiologists (blind to patients’ medical history, 8、10 、20 years of experience in diagnostic chest radiology) independently reviewed and staged the CT findings. In cases of disagreement, a consensus was reached through consultation and discussion. CT analysis included large nodules (diameter > 10 mm), consolidation, cavitary lesions, number of cavitary lesions, diameter of the largest cavitary lesion, maximum wall thickness of the largest cavitary lesion, cavitary lesion with fluid-air interfaces, bronchial dilatation, exudative lesions(inflammation of the air spaces and/or interstitium. Patchy, ground glass, or flocculent clouding is seen in the lungs bilaterally or unilaterally.), fibrous lesions(consisting of a fibrous texture, are a healing feature of acute and chronic pneumonia and appear mainly as irregular strips or reticular clouding on CT scans.), enlarged hilar or mediastinal lymph nodes, and calcification of hilar or mediastinal lymph nodes.

Pulmonary TB was also classified as mild, moderate, or severe according to the chest CT imaging findings [4].

### CT imaging classification criteria

Mild: No cavitary lesions, only part of one or both lungs are involved, and the entire extent is less than the volume of the lung on the side above the junction of the second rib and the sternum.

Moderate: Lesions located in one or both lungs, but the entire extent does not exceed any of the following: (1) small or moderate diffuse lesions distributed over no more than the entire area of one lung. If the lesions are bilateral, the total area of the lesion does not exceed the area of one lung; (2) a high-density fused lesion with a density not exceeding one-third of the volume of one lung; or (3) a cavitary lesion with a maximum diameter of less than 4 cm.

**Fig. 1:**
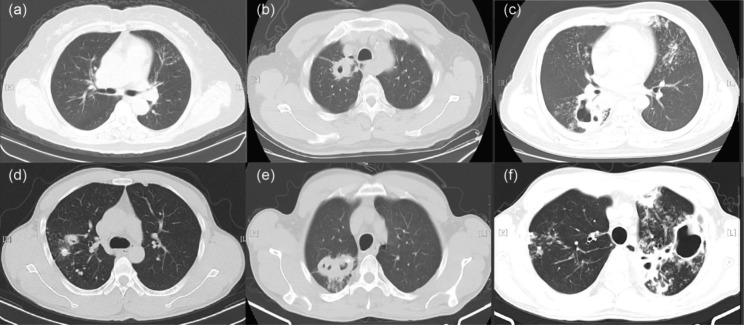
Representative chest CT images of mild, moderate, and severe cases in the TB-DM and TB-NDM groups. (**a**) Mild CT image of the TB-NDM. (**b**) CT imaging of moderate TB-NDM. (**c**) CT imaging of severe TB-NDM. (**d**) CT imaging of mild TB-DM. (**e**) CT imaging of moderate TB-DM. (**f**) CT imaging of severe TB-DM. CT: Computed tomography, PTB: Pulmonary Tuberculosis, TB-DM: TB and comorbid T2DM, TB-NDM: TB without comorbid T2DM

Severe: Lesions are more extensive than a moderate lesion.(Fig. 1).

### Statistical analysis

Student’s t-tests were used to assess the differences in the distribution of demographic characteristics between cases and controls, the Wilcoxon signed-rank test was used to analyse discontinuous variables, Fisher’s exact test was used to analyse binary categorical variables, and the chi-square test was used to analyse the trend for multi-category categorical variables. GraphPad 8 software was used for all statistical analyses. We used Bonferroni correction to control for multiple comparisons, and the P-value threshold was set at 0.05.

## Results

### Baseline characteristics

**Fig. 2:**
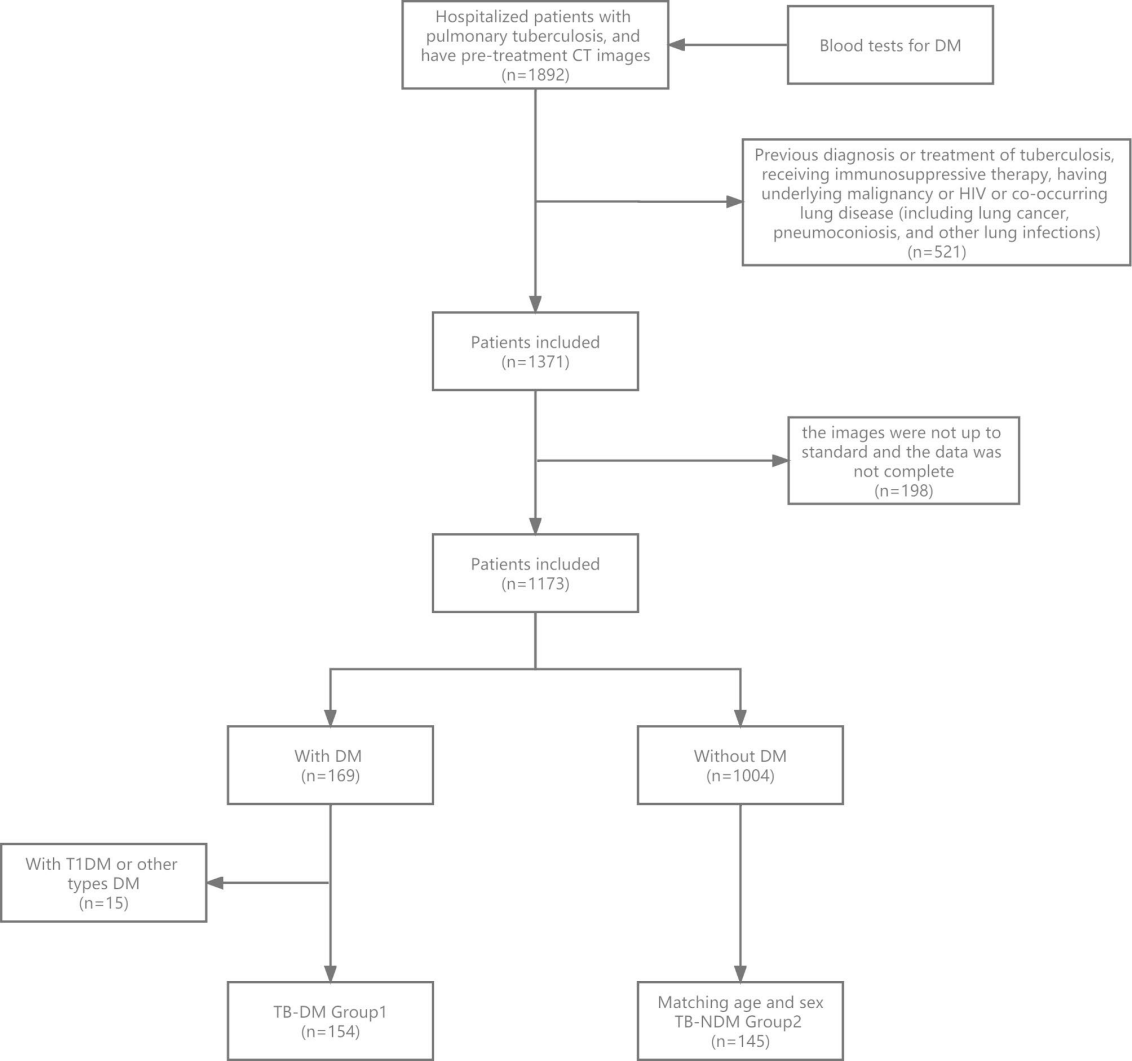
Flowchart for patients’ enrollment. CT: Computed tomography; TB: Tuberculosis; HIV: Human Immunodeficiency Virus; DM: Diabetes Mellitus; CT: Computed tomography; T1DM: Type 1 diabetes mellitus; T2DM: Type 2 diabetes mellitus

Records of 1892 patients hopsitalised for active pulmonary TB were reviewed. A total of 299 patients with pulmonary TB (154 in the TB-DM group and 145 in the TB group) were included in the study **(**Fig. 2). The clinical characteristics of the two groups are summarised in Table 1. The patients in the two groups were age- and sex-matched, and no significant group differences were noted in terms of febrile symptoms, history of smoking, history of alcohol consumption, or incidence of extrapulmonary TB, which was diagnosed according to the National Health and Family Planning Commission of the People’s Republic of China, Health Industry Standard of the People’s Republic of China - Classification of Tuberculosis guidelines. No significant difference was noted in the overall drug resistance between the two groups (eight patients in each group). The TB-DM group included cases of rifampicin resistance, whereas the TB-NDM group included cases of rifampicin, ethambutol, and isoniazid resistance.

**Table 1:** Clinical characteristics in PTB patients with or without T2DM

Characteristic	TB-DM(n = 154)	TB-NDM(n = 145)	P-value	Adjusted P value^#^
**Demographic characteristic**				
Age,year(mean$\pm$SD)	53.58$\pm$13.77	52.80$\pm$13.94	0.6291	> 0.9999
Sex(male,n,%)	115(74.68)	109(75.17)	> 0.9999	> 0.9999
Smoking(n,%)	68(44.16)	77(53.10)	0.1332	> 0.9999
Drinking(n,%)	60(38.96)	50(34.48)	0.4720	> 0.9999
Extra-pulmonary tuberculosis(n,%)	18(11.69)	21(14.48)	0.4965	> 0.9999
**Clinical symptoms(n,%)**				
Cough and sputum	140(90.90)	132(91.03)	> 0.9999	> 0.9999
Fever	62(40.26)	40(27.59)	0.0278^*^	0.3614
Chest pain	25(16.23)	19(13.10)	0.5146	> 0.9999
Weight loss、Hemoptysis、Headaches, etc.	57(37.01)	69(47.59)	0.0991	> 0.9999
**Drug-resistant TB(n,%)**				
Rifampicin	8(5.19)	5(3.45)	0.5748	> 0.9999
Ethambutol	0(0.00)	2(1.38)	0.2343	> 0.9999
Isoniazide	0(0.00)	1(0.69)	0.4849	> 0.9999

^#^Bonferroni correction is used to control for multiple comparisons, and the adjusted p-value is obtained by multiplying the observed (unadjusted) p-value with the number of comparisons

PTB: Pulmonary Tuberculosis, T2DM: Type 2 diabetes mellitus, DM-TB: TB and comorbid T2DM, TB-NDM: TB without comorbid T2DM,*: P<0.05

### Characteristics of CT findings

**Fig. 3:**
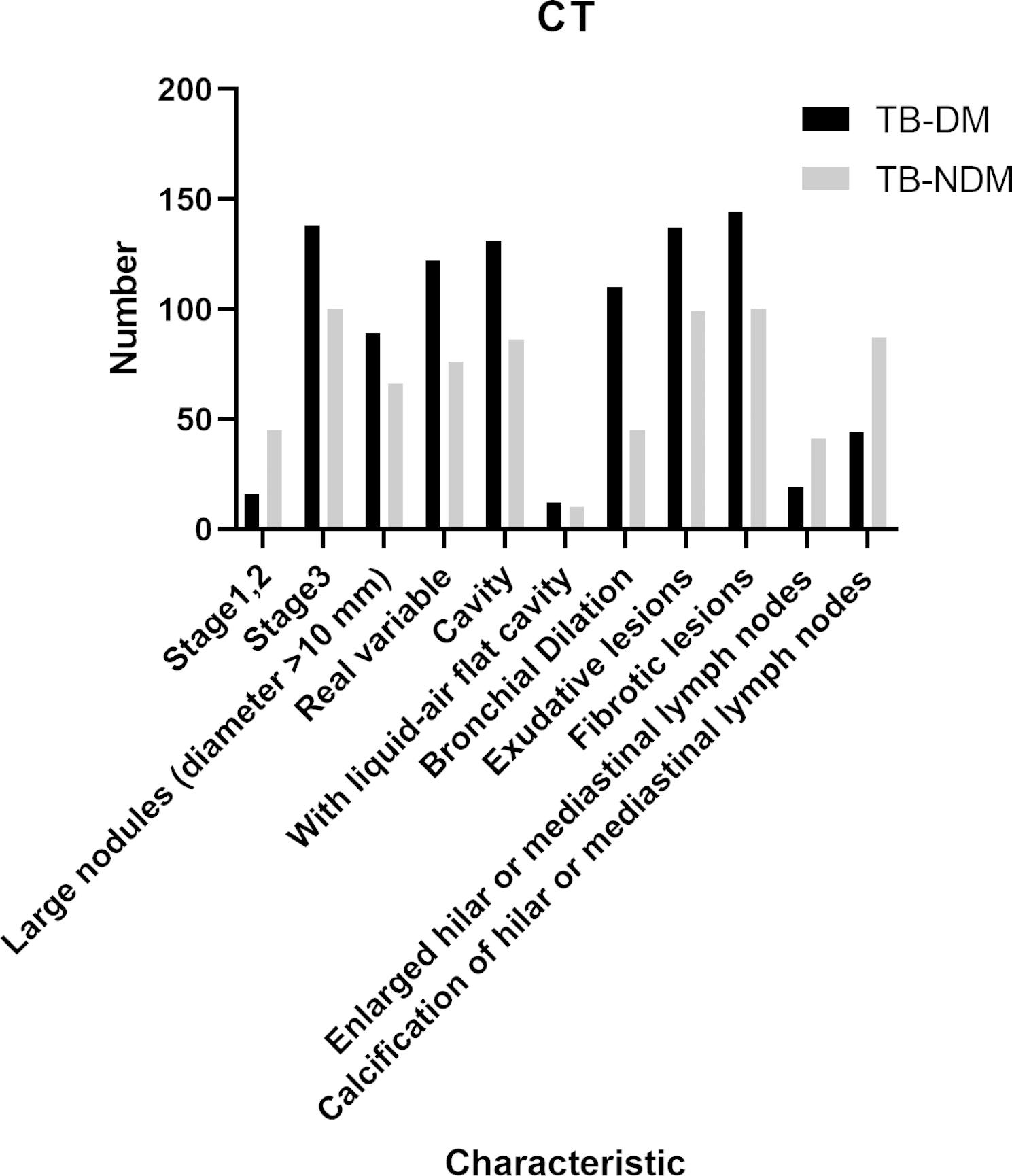
Number of patients with various typical lung CT signs in TB-DM and TB-NDM groups. CT: Computed tomography,PTB:Pulmonary Tuberculosis, TB-DM:TB and comorbid T2DM,TB-NDM:TB without comorbid T2DM

Cases of pulmonary TB were classified as mild, moderate, or severe based on CT imaging classification criteria, and patients were divided into two groups: a mild-to-moderate group and a severe group. There were significantly more patients with severe TB in the TB-DM group than in the TB-NDM group (P < 0.0001), and significant differences were noted in lesion site (P = 0.0133) and the number of lung lobes involved (P < 0.0001) between patients with TB-DM and those with TB-NDM. The CT characteristics of the two groups of patients are shown in Fig. 3 and Table 2.

**Table 2:** Analysis of chest CT images in patients with PTB in DM and NDM group

Characteristic	TB-DM (n = 154)	TB-NDM (n = 145)	P value	Adjusted P value^#^
Stage(n,%)			^****^	^****^
Mild/Moderate(n,%)	16(10.39)	45(31.03)	-	-
Severe(n,%)	138(89.61)	100(68.97)	-	-
Location (lobe n,%)			0.0133^*^	0.1729
Upper right(n,%)	36(23.38)	44(30.34)	-	-
Middle right(n,%)	19(12.34)	32(22.07)	-	-
Lower right(n,%)	28(18.18)	32(22.07)	-	-
Upper left(n,%)	37(24.03)	39(26.90)	-	-
Lower left(n,%)	27(17.53)	23(15.86)	-	-
Total(n,%)	103(66.88)	80(55.17)	-	-
Large nodules (diameter > 10 mm)(n,%)	89(57.79)	66(45.52)	0.0375^*^	0.4875
Consolidation(n,%)	122(79.22)	76(52.41)	^****^	^****^
Cavity(n,%)	131(85.06)	86(59.31)	^****^	^****^
Median Number of cavities(1–5)	4	2	^****^	^****^
Maximum cavity diameter (mm)^^^	49.93$\pm$22.48	48.29$\pm$22.40	0.6007	> 0.9999
Maximum cavity wall thickest place (mm)^^^	18.13$\pm$11.61	9.72$\pm$4.99	^****^	^****^
Cavity with air-fluid level(n,%)	12(9.16)	10(11.63)	0.8271	> 0.9999
Bronchial Dilation(n,%)	110(71.43)	45(31.03)	^****^	^****^
Exudative lesions(n,%)	137(88.96)	99(68.28)	^****^	^****^
Fibrotic lesions(n,%)	144(93.51)	100(68.97)	^****^	^****^
Enlarged hilar or mediastinal lymph nodes(n,%)	19(12.34)	41(28.28)	0.0008^***^	0.0104^*^
Calcification of hilar or mediastinal lymph nodes(n,%)	44(28.57)	87(60.00)	^****^	^****^

^#^Bonferroni correction was used to control for multiple comparisons, and the adjusted p-value is obtained by multiplying the observed (unadjusted) p-value with the number of comparisons

^^^Mean±SD

CT: Computed tomography, PTB: Pulmonary Tuberculosis, TB-DM: TB and comorbid T2DM,TB-NDM: TB without comorbid T2DM,*: P < 0.05, ***: P < 0.001, ****: P < 0.0001

Patients with TB-DM had a higher proportion of consolidation (79.22% vs. 52.41%, P < 0.0001), cavitary lesions (85.06% vs. 59.31%, P < 0.0001), bronchiectasis (71.43% vs. 31.03%, P < 0.0001), and exudative lesions (88.96% vs. 68.28%, P < 0.0001) than patients with TB-NDM. Patients with TB-NDM had a higher proportion of enlarged and calcified hilar mediastinal lymph nodes (28.28% vs. 12.34%, P = 0.0008) (60.00% vs. 28.57%, P < 0.0001) than patients with TB-DM. The number of cavitary lesions was significantly higher in patients with TB-DM than in those with TB-NDM (P < 0.0001), and the maximum thickness of the wall of the largest cavitary lesion was also significantly greater in patients with TB-DM than in patients with TB-NDM (18.13 ± 11.61 mm vs. 9.72 ± 4.99 mm, P < 0.0001). There were no significant differences in the distribution of lesions in the lung lobes between TB-DM and TB-NDM patients.

## Discussion

The present study focused on the clinical and CT manifestations of pulmonary TB and T2DM in hospitalised patients from the LiangShan Yi regions. In recent years, the prevalence of pulmonary TB has increased in rural areas of China, especially in western China, these patients are more likely to ignore persistent cough and delay seeking care [15]. At the same time, the burden of non-communicable diseases such as diabetes is increasing rapidly in low- and middle-income countries and the risk of T2DM is higher among low-income populations [16]. For low-income patients, TB-DM not only has a greater health impact but also poses greater challenges to their economic situation.

Studies [8–13] suggest that diabetes affects the clinical features and CT manifestations of patients with TB, but the exact outcome of this effect remains to be elucidated. Few studies [17, 18] have reported more severe clinical symptoms in patients with pulmonary TB when comorbid with diabetes but the exact mechanism remains unclear. No significant differences in clinical symptoms were found in our study, possibly because many patients had already experienced a long disease course and had more symptoms at presentation which may have been due to a lack of medical resources in the region.

CT is a common diagnostic tool for TB. We found that significantly more patients with TB-DM had severe TB, as judged by CT, than those with TB-NDM. Patients with TB-DM were more likely to have cavitary lung lesions, especially thick-walled cavities, as well as severe consolidation and more lung lobes involved than those with TB-NDM, as corroborated by other studies [19, 20]. Moreover, some studies [8, 13] have shown that single or multiple nodules or masses and exudative lesions were more common with increased blood glucose, which is similar to the results of the present study.

The mechanism of more extensive and severe pulmonary lesions in patients with TB-DM may be related to the hyperglycaemic, immune-compromised internal environment of diabetes. First, insulin resistance in patients with T2DM leads to elevated blood glucose and increased degradation of adipose tissue, which together create an internal environment conducive to the survival and reproduction of *Mycobacterium tuberculosis* [8]. Second, hyperglycaemia leads to persistent inflammation. Owing to impaired innate immunity, hyperglycaemia, and oxidative stress together induce elevation in the level of pulmonary cytokines such as interleukin (IL)-17 A, IL-8, and IL-10, which leads to increased pro-inflammatory responses [21, 22]. In contrast, interferon-gamma production is decreased due to Th1 cell impairment, leading to the inability to control the growth and multiplication of *M. tuberculosis* [21]. Third, hyperglycaemia suppresses immune function, leading to delayed antigen presentation and impaired bactericidal activity and promoting immune escape by *M. tuberculosis* [8]. High bacterial load and excessive inflammation lead to more extensive pulmonary lesions, cavitation, and immune cell infiltration [23]. Fourth, high glucose levels enhance pathological signalling associated with hypercoagulation caused by TB infection and promote systemic activation of coagulation, a process that leads to the exacerbation of caseous necrosis in granulomas with severe fibroplasia [24]. This mechanism may explain the increase in fibrous lesions in the TB-DM group in the present study [25].

There are many studies [8, 9, 17, 26] suggesting that TB-DM patients have lesions primarily in the lower lungs and involving more lobes. In contrast, our results showed no statistical difference between the TB-DM and DM groups in terms of the location of the TB lesions. We speculate that the differences between the lobes were not statistically significant due to the longer duration and more severe disease of the patients, resulting in a wider distribution of pulmonary lesions. In addition, the statistical method we used in this study may have also affected the significance of our findings, as the Bonferroni correction is a conservative statistical method, and further studies with larger samples are desired.

Some studies have reported that lymph node lesions are more common in patients with TB-DM than in TB-NDM patients [27, 28], while our findings showed that the proportion of enlarged and calcified hilar and mediastinal lymph nodes was significantly higher in patients with TB-NDM than in the TB-DM group (28.57% vs. 60.00%). Lymph nodes are not only sites of antigen presentation and immune activation during infection, but may also be important sites for the persistence of large numbers of M tuberculosis, and TB may persist in and be transmitted through lymph nodes [29]. However, it has also been argued [30] that TB may be considered a lymphatic disease and a lymphatic model of TB has been proposed, which states that many patients have enlarged lymph nodes that might have been noted merely as an ancillary observation, rather than as a key reservoir of tuberculosis infection, which is more consistent with our results and could be studied in the future by expanding the sample size.

The present study has some limitations. First, this was a cross-sectional study, and although the role of CT in helping diagnose pulmonary TB in patients with T2DM is suggested, we could not establish a causal relationship between T2DM and severe radiological manifestations. Second, this was a single-centre study with a small sample size; therefore, large-sample, multi-centre studies are required in the future. Finally, this was an observational study, and potential selection bias in the study cohort may be unavoidable. In this study, we investigated the differences in clinical features and CT signs in patients with TB-DM and observed an increase in pulmonary lesions in patients with T2DM, suggesting that timely diagnosis, detection, and treatment of diabetes may help control pulmonary TB. Meanwhile, the subjects of this study were people in deprived areas. As we all know, because of economic and cultural factors, and medical conditions, rural areas face a more serious situation of diagnosis and treatment of TB. CT can promptly find characteristic manifestations in the lungs of patients with TB-DM.

## Conclusions

Among patients with pulmonary TB, those with comorbid T2DM had more severe chest CT findings, more cavitary lesions larger in size and with thicker walls, more bronchial dilatation, and more consolidation, exudative lesions, and fibrous lung lesions. There were no significant differences in the distribution of lesions in the lung lobes between TB-DM and TB-NDM patients and the clinical manifestations were not significantly different between the two groups. This study is the first to report the imaging characteristics of chest CT in patients with active pulmonary TB complicated by T2DM in a rural area. This study may help local authorities analyse the factors related to TB comorbid with T2DM and formulate countermeasures and measures for disease control or health promotion.

## Electronic supplementary material

Below is the link to the electronic supplementary material.

Supplementary Material 1

## Data Availability

The datasets generated during and analyzed during the current study are not publicly available due to privacy but are available from the corresponding author on reasonable request.

## Abbreviations

CT: Computed tomography
IL: Interleukin
T2DM: Type 2 diabetes mellitus
TB: Tuberculosis
TB-DM: TB and comorbid T2DM
TB-NDM: TB without comorbid T2DM
