# Supplementary material for: Computed tomography findings in patients with pulmonary tuberculosis and diabetes at an infectious disease hospital in China: a retrospective cross-sectional study
Source: BMC Infect Dis. 2023 Jun 27;23:436. doi: 10.1186/s12879-023-08386-7 (PMC10304231; doi:10.1186/s12879-023-08386-7)
Supplement: Supplementary file 1 — Supplementary Material 1 [file 12879_2023_8386_MOESM1_ESM.docx]

**Supplemental Appendix**

1、Exudative lesions: i.e., inflammation of the air spaces and/or interstitium. Patchy, ground glass, or flocculent clouding is seen in the lungs bilaterally or unilaterally.


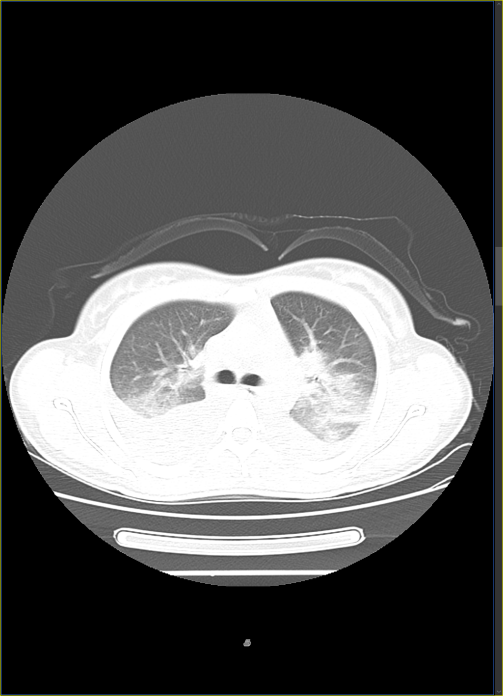


2、Fibrous lesions: consisting of a fibrous texture, are a healing feature of acute and chronic pneumonia and appear mainly as irregular strips or reticular clouding on CT scans.


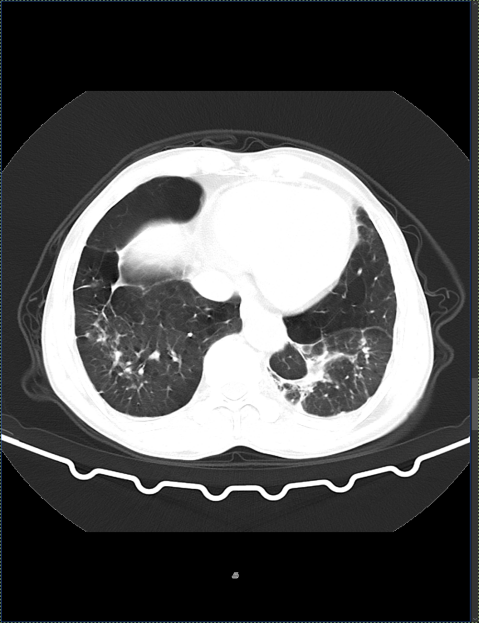


3、Consolidation lesions :those in which the alveolar cavity is replaced by fluid, blood, pus, cells, or other material. On CT, the density is higher than that of the lung tissue, masking the lung texture, and the density is more uniform.


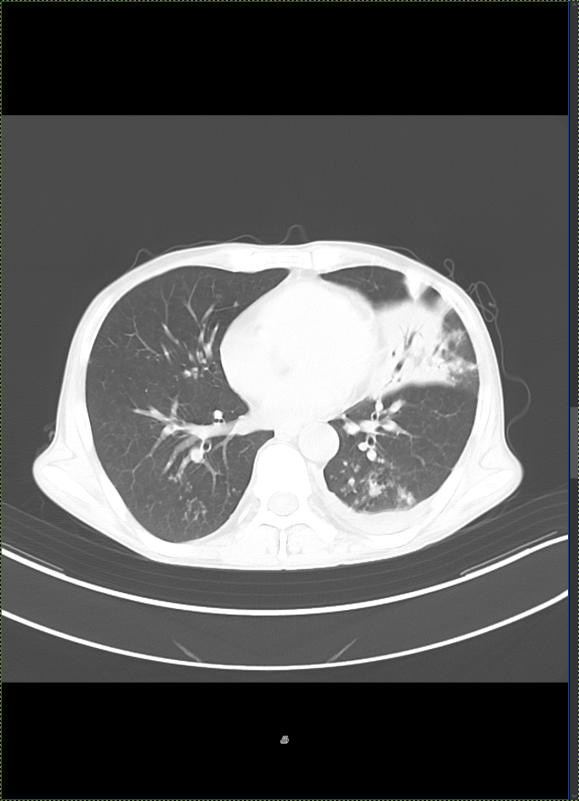

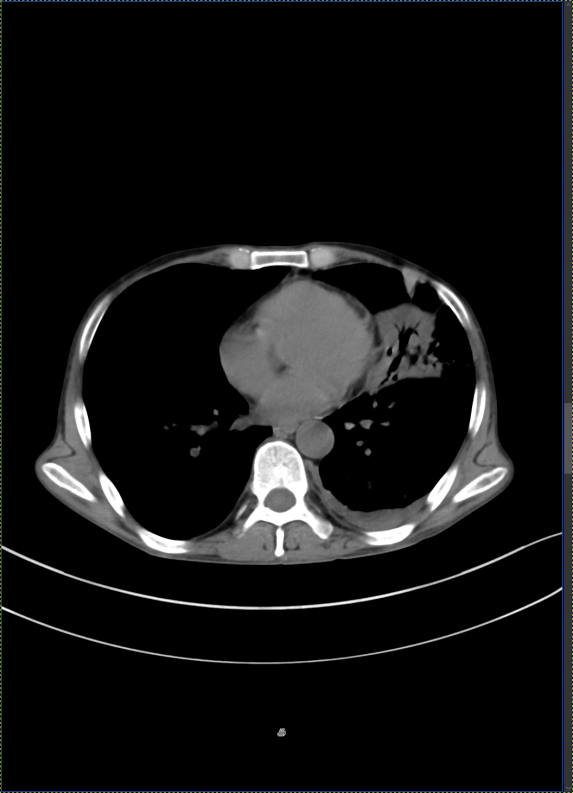


4、Mild: No cavitary lesions, only part of one or both lungs are involved, and the entire extent is less than the volume of the lung on the side above the junction of the second rib and the sternum.

Moderate: Lesions located in one or both lungs, but the entire extent does not exceed any of the following: 1) small or moderate diffuse lesions distributed over no more than the entire area of one lung. If the lesions are bilateral, the total area of the lesion does not exceed the area of one lung; 2) a high-density fused lesion with a density not exceeding one-third of the volume of one lung; or 3) a cavitary lesion with a maximum diameter of less than 4 cm.

Severe: Lesion is more extensive than a moderate lesion.


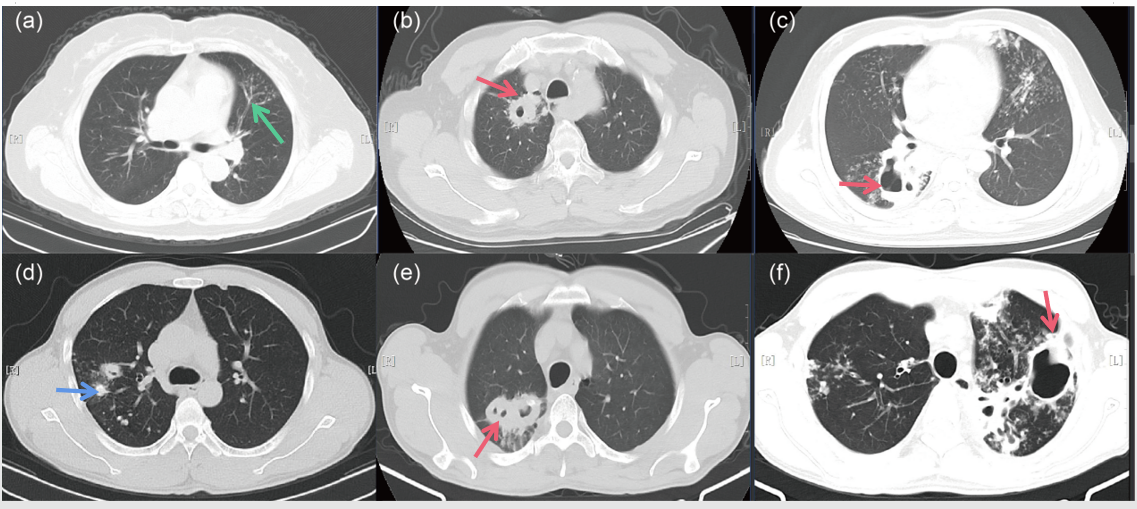


Red arrows show cavities, green arrows show exudative lesions, and blue arrows show nodules.

Mild, moderate, and severe chest CT representative images in TB-DM and TB-NDM group.(a)Mild CT imaging in TB-NDM.(b)Moderate CT imaging in TB-NDM.(c)Severe CT imaging in TB-NDM.(d)Mild CT imaging in TB-DM.(e)Moderate CT imaging in TB-DM.(f)Severe CT imaging in TB-DM.
